# When the Heat Is On: The Effect of Temperature on Voter Behavior in Presidential Elections

**DOI:** 10.3389/fpsyg.2017.00929

**Published:** 2017-06-08

**Authors:** Jasper Van Assche, Alain Van Hiel, Jonas Stadeus, Brad J. Bushman, David De Cremer, Arne Roets

**Affiliations:** ^1^Department of Developmental, Personality, and Social Psychology, Ghent UniversityGhent, Belgium; ^2^School of Communication and Department of Psychology, The Ohio State University, ColumbusOH, United States; ^3^Judge Business School, University of CambridgeCambridge, United Kingdom

**Keywords:** excitation transfer theory, presidential elections, prosocial behavior, temperature, voter turnout, voting result

## Abstract

Hot temperatures lead to heightened arousal. According to excitation transfer theory, arousal can increase both antisocial and prosocial behavior, depending on the context. Although many studies have shown that hot temperatures can increase antisocial behavior, very few studies have investigated the relationship between temperature and prosocial behavior. One important prosocial behavior is voting. We analyzed state-level data from the United States presidential elections (*N* = 761). Consistent with excitation transfer theory, which proposes that heat-induced arousal can transfer to other activities and strengthen those activities, changes in temperature and voter turnout were positively related. Moreover, a positive change in temperature was related to a positive change in votes for the incumbent party. These findings add to the literature on the importance of non-ideological and non-rational factors that influence voting behavior.

## Introduction

Hot temperatures can have divergent effects on human behavior (Oishi, 2014). On the one hand, ample studies have shown that hot temperatures have been associated with antisocial behaviors (e.g., assaults, murders; Anderson and Bushman, 2002; Bushman et al., 2005) as well as negative political behaviors, such as political rebellions, and riots (Lombroso, 1911; Schwartz, 1968; Carlsmith and Anderson, 1979). On the other hand, hot temperatures have been associated with prosocial behaviors (e.g., helping, leaving more generous tips; Cunningham, 1979; Guéguen and Lamy, 2013), while no studies have investigated potential temperature effects for positive political behaviors yet.

Excitation transfer theory (Zillmann, 2003) explains these divergent effects in terms of the arousal invoked by hot temperatures. Arousal involves the activation of the autonomic nervous and endocrine systems, leading to an increased heart rate and blood pressure and a condition of sensory alertness, mobility, and readiness to respond behaviorally. Specifically, the undesignated arousal created by high temperatures can be misattributed to another stimulus, which intensifies the individual’s emotional response to this other stimulus (Zillmann, 2003). As a result, arousal makes negative experiences even more negative, as such facilitating aggression (Anderson and Bushman, 2002). Conversely, positive experiences become even more positive, which leads people to behave as good Samaritans and exemplary citizens (Foster et al., 1998).

As such, increases in arousal due to increases in temperature might impact the result of an election, because of its proposed impact on collective behaviors such as voter turnout. Such positive collective behavior, however, has received little to no empirical attention, and the present study helps to fill this gap in the literature by investigating the relation between temperatures and voting behavior. Excitation transfer theory predicts that heat increases arousal, and that this arousal mobilizes people to take action — including political action such as voting. This study investigated how *changes* in temperature (rather than absolute temperature)^1^ relate to *changes* in voting behavior. For example, an absolute temperature of 30°C (86°F) is a normal temperature in California, but is very hot in Alaska. Moreover, absolute temperature is related to many other variables confounded with temperature. For example, poverty is generally higher in countries with hotter climates. Hence, by studying change variables *within* geographically defined entities, the variation *between* these entities on a number of confounding variables is minimized.

## The Present Study

We analyzed the relationship between temperature and voting using data from presidential elections from 1960 to 2016 in each state in the United States. In addition to mere voter turnout, this study also investigated how hot temperatures may pose costs as well as benefits for different political parties. Specifically, previous studies have found that hot temperatures increase anger (Anderson and Bushman, 2002; Bushman et al., 2005), which, in turn, motivates people to vote (Valentino et al., 2011; Van Zomeren, 2016). We predicted that such non-rational influence costs more votes for the incumbent party than for alternative parties. When people are angry with the current state of the country, they may choose to vote for a new candidate who promises a change. For example, one study found that parties that emphasize system change are especially likely to benefit from anger-based voting (Van Zomeren et al., 2016). In contrast, temperature-related positive emotions should gain more votes for the incumbent party than for alternative parties because people are happy with the current state of the country. For example, one study found that citizens who were interviewed on sunny days reported the highest levels of satisfaction with democracy, the government, and the economy (Mutz and Kampfer, 2011).

## Methods

We collected data from United States presidential elections from 1960 to 2016 in each state (and in Washington, DC, United States). We chose 1960 as the starting date because only from 1960 onward voter turnout per state was electronically available. The temperature data were retrieved from the web application of the National Centers for Environmental Information. We selected a weather station close to the center of population for each state. Moreover, the selected stations should have data that go back to 1960 and should have a high degree of coverage, i.e., few missing data. In case of missing data, we sought the nearest station, and in all these cases data was available from a neighboring station within a close distance. Not only maximum temperature on Election Day was retrieved, but also the maximum temperature of the 7 days preceding the elections was collected, and we calculated the relative change with regards to the previous Election Day. A full description of the multilevel methods and assessment in state level temperature changes can be found in the Supplementary Materials.

Note that some election studies used more fine-grained geographical entities such as the county or municipality level to analyze the effects of precipitation, which is warranted because precipitation may show much local variation. On the other hand, temperature (and changes herein) is rather stable (e.g., Kusuda and Achenbach, 1965), and the inclusion of more fine-grained entities would unnecessarily inflate the effect of geographical entity. As most election studies with American samples (e.g., Geys, 2006), voter turnout was calculated by the following formula: 100% – [(Voting Age Population [VAP] – number of votes)/VAP].

The study included 761 data points. Multilevel modeling (MLM) with election dates (individual level) nested within states (contextual level) was conducted. A *random intercept model* that allowed intercept coefficients to vary across states was used (Raudenbush and Bryk, 2002). We constructed four similar models, one for predicting change in voter turnout, and three for changes in votes for the non-system parties (i.e., Greens, Independents, Libertarians), the challenger party (when a Democrat president has been in office during the last 4 years, the Republican party is the challenger party, and vice versa), and the incumbent party. We controlled for nine variables relevant to voting behavior (see also Curriero et al., 2002): (1) latitude and (2) longitude of the most populated area of each state, (3) maximum temperature on Election Day, (4) mean temperature the week before Election Day, (5) the president being available for reappointment, (6) the incumbent president being elected or not (Presidents Lyndon Johnson and Gerald Ford were vice presidents), (7) presidential approval ratings, (8) whether the president’s party had a majority in Congress during the two last years, and (9) change in state gross domestic product (GDP). A full description of and justification for these control variables can be found in the Supplementary Materials.

## Results

In the model predicting voter turnout, we included the control variables in the first block and added change in temperature compared to the previous election in the second block. In the models predicting change in voting results, we constructed similar multilevel models but added change voter turnout in the third block. **Table 1** shows the unstandardized estimates of the multilevel regression analyses on the respective outcomes when no control variables were included. As can be seen in **Tables 2, 3**, relationships remained significant even when control variables were included. A positive change in temperature on Election Day remained significantly related to an increase in voter turnout. For each increase of 1°C (1.8°F), voter turnout increased by 0.14%.

**Table 1:** Unstandardized estimates (standard errors in parentheses) of multilevel hierarchical regression analyses on change in voter turnout and voting result.

	Voter turnout	Non-system parties	Challenger party	Incumbent party
	
Predictor	*b (SE)*	*b (SE)*	*b (SE)*	*b (SE)*
Δ Temperature on election day	0.37^∗∗∗^ (0.05)	–0.19^∗∗∗^ (0.06)	–0.12^∗^ (0.05)	0.29^∗∗∗^ (0.06)
*Δ R2 (%)*	*8.16*^∗∗∗^	*1.49*^∗∗∗^	*2.46*^∗^	*3.75*^∗∗∗^
Δ Voter turnout		0.33^∗∗∗^ (0.04)	–0.05 (0.04)	–0.29^∗∗∗^ (0.05)
*Δ R2 (%)*		*7.31*^∗∗∗^	*1.23*	*2.09*^∗∗∗^

*^∗^*p* < 0.05; ^∗∗∗^*p* < 0.001.*

**Table 2:** Unstandardized estimates (standard errors in parentheses) of multilevel hierarchical regression analyses on change in voter turnout.

	Step 1	Step 2
	
Predictor	*b (SE)*	*b (SE)*
Latitude	0.04 (0.08)	–0.03 (0.09)
Longitude	0.00 (0.01)	0.00 (0.01)
Temperature Election Day	0.14^∗^ (0.06)	0.00 (0.08)
Temperature week before Election Day	0.06 (0.09)	0.12 (0.09)
President eligible for reappointment (1 = yes)	–1.43^∗∗∗^ (0.31)	–1.40^∗∗∗^ (0.31)
President elected (1 = yes)	0.15 (0.41)	0.06 (0.41)
Approval rating incumbent president	0.13^∗∗∗^ (0.03)	0.13^∗∗^ (0.04)
Majority in congress in last 2 years (1 = yes)	1.55^∗∗∗^ (0.31)	1.47^∗∗^ (0.31)
Δ State GDP per capita	–18.54^∗∗∗^ (3.28)	–18.30^∗∗∗^ (3.27)
Δ Temperature on Election Day		0.14^∗^ (0.06)
*Δ R2 (%)*	*41.08*^∗∗∗^	*0.85*^∗^

*GDP, gross domestic product; ^∗^*p* < 0.05; ^∗∗^*p* < 0.01; ^∗∗∗^*p* < 0.001.*

**Table 3:** Unstandardized estimates (standard errors in parentheses) of multilevel hierarchical regression analyses on change in votes for alternative parties (independent, libertarians, greens), the challenger mainstream party and the incumbent party.

	Non-system parties			Challenger mainstream party			Incumbent party		
	Step 1	Step 2	Step 3	Step 1	Step 2	Step 3	Step 1	Step 2	Step 3
	
Predictor	*b* (*SE*)	*b (SE)*	*b (SE)*	*b (SE)*	*b (SE)*	*b (SE)*	*b (SE)*	*b (SE)*	*b (SE)*
Latitude	–0.23 (0.13)	0.00 (0.14)	0.01 (0.13)	–0.34^∗∗∗^ (0.11)	–0.34^∗∗^ (0.11)	–0.34^∗∗^ (0.11)	0.61^∗∗∗^ (0.14)	0.38^∗∗^ (0.14)	0.36^∗∗^ (0.14)
Longitude	0.01 (0.02)	0.01 (0.02)	0.01 (0.02)	0.04^∗^ (0.02)	0.04^∗^ (0.02)	0.04^∗^ (0.02)	–0.04^a^ (0.02)	–0.05^∗^ (0.02)	–0.05^∗^ (0.02)
Temperature Election Day	–0.25^∗∗^ (0.10)	0.18 (0.13)	0.18 (0.12)	0.07 (0.08)	0.08 (0.11)	–0.08 (0.11)	0.20^∗^ (0.10)	–0.22^a^ (0.13)	–0.22^a^ (0.13)
Temperature week before	–0.03 (0.14)	–0.21 (0.14)	–0.26^a^ (0.14)	–0.43^∗∗∗^ (0.11)	–0.43^∗∗∗^ (0.12)	–0.43^∗∗∗^ (0.12)	0.45^∗∗^ (0.15)	0.63^∗∗∗^ (0.15)	0.68^∗∗∗^ (0.14)
President eligible for reappointment	–1.70^∗∗∗^ (0.47)	–1.80^∗∗∗^ (0.46)	–1.22^∗∗^ (0.45)	–2.55^∗∗∗^ (0.38)	–2.55^∗∗∗^ (0.38)	–2.60^∗∗∗^ (0.39)	4.32^∗∗∗^ (0.49)	4.42^∗∗∗^ (0.48)	3.85^∗∗∗^ (0.48)
President elected	0.22 (0.62)	0.51 (0.62)	0.48 (0.59)	2.82^∗∗∗^ (0.51)	2.82^∗∗∗^ (0.51)	2.83^∗∗∗^ (0.51)	–3.16^∗∗∗^ (0.66)	–3.44^∗∗∗^ (0.65)	–3.42^∗∗∗^ (0.63)
Approval rating	–0.11^∗^ (0.05)	–0.04 (0.05)	–0.08 (0.05)	–0.29^∗∗∗^ (0.04)	–0.28^∗∗∗^ (0.04)	–0.28^∗∗∗^ (0.04)	0.40^∗∗∗^ (0.06)	0.32^∗∗∗^ (0.06)	0.37^∗∗∗^ (0.05)
Majority in Congress	3.93^∗∗∗^ (0.48)	4.17^∗∗∗^ (0.47)	3.56^∗∗∗^ (0.46)	–0.50 (0.39)	–0.50 (0.39)	–0.46 (0.40)	–3.58^∗∗∗^ (0.50)	–3.82^∗∗∗^ (0.50)	–3.22^∗∗∗^ (0.49)
Δ State GDP per capita	–4.20 (5.00)	–4.97 (4.91)	2.62 (4.83)	–10.60^∗∗^ (4.08)	–10.60^∗∗^ (4.09)	–11.08^∗∗^ (4.18)	14.72^∗∗^ (5.26)	15.48^∗∗^ (5.18)	8.02 (5.12)
Δ Temperature on Election Day		–0.43^∗∗∗^ (0.09)	–0.49^∗∗∗^ (0.08)		0.00 (0.07)	0.00 (0.07)		0.43^∗∗∗^ (0.09)	0.48^∗∗∗^ (0.09)
Δ Voter turnout			0.42^∗∗∗^ (0.06)			–0.03 (0.05)			–0.41^∗∗∗^ (0.06)
*Δ R2 (%)*	*6.37*^∗∗∗^	*3.75*^∗∗∗^	*7.65*^∗∗∗^	*17.29*^∗∗∗^	*0.00*	*0.04*	*15.97*^∗∗∗^	*3.26*^∗∗∗^	*6.62*^∗∗∗^

*GDP, gross domestic product; ^a^*p* < 0.05, one tailed; ^∗^*p* < 0.05; ^∗∗^*p* < 0.01; ^∗∗∗^*p* < 0.001.*

**Tables 2, 3** also reveal that changes in temperature were related to both voter turnout and voting results. We therefore used bootstrap analyses (50,000 bootstrap samples) to decompose the total temperature effect into an indirect effect of temperature through voter turnout on voting result, as well as a direct effect of temperature on voting result. This analysis revealed significant indirect effects that corroborate the hypothesis that temperature-based increases in turnout are motivated by voters who want political change, and this at the cost of the incumbent party (*b* = 0.14^∗^0.42 = 0.06; Boot *SE* = 0.03; CI_95_ = [0.01, 0.13]; *z* = 2.23, *p* = 0.026, for the non-system parties; *b* = 0.14^∗^–0.41 = –0.06; Boot *SE* = 0.03; CI_95_ = [–0.12, –0.01]; *z* = –2.21, *p* = 0.027, for the incumbent party)^2^. The indirect effect was non-significant for the challenger party (*b* = 0.14^∗^–0.03 = 0.00; Boot *SE* = 0.01; CI_95_ = [–0.04, 0.01]; *z* = –0.48, *p* = 0.632). However, the direct, unmediated effect of temperature on change in votes ran in the opposite direction, as it was significantly negative for the alternative parties (*b* = –0.49, *p* < 0.001), significantly positive for the incumbent party (*b* = 0.48, *p* < 0.001), and non-significant for the challenger party (*b* = 0.00). In other words, although positive changes in temperature motivate some citizens to cast their votes for the non-system parties, they are an even stronger motivator for some citizens to vote for the incumbent government.

## Discussion

Previous studies have shown that hot temperatures are related to negative collective behavior (Lombroso, 1911; Schwartz, 1968; Carlsmith and Anderson, 1979), whereas the present study offers a first demonstration in the literature that changes in temperature are related to positive collective behavior (i.e., an increase in democratic and non-violent political behavior in the form of voter turnout). This result adds to the literature as former studies exclusively investigated temperature effects on violent mass behavior (Lombroso, 1911; Schwartz, 1968; Carlsmith and Anderson, 1979). This result is also in line with excitation transfer theory (Zillmann, 2003), which holds that temperature effects are modulated by the context, and explains why voter turnout is facilitated in the context of high profile presidential elections, whereas aggressive behaviors are facilitated in the context of mass protest and revolt.

Moreover, the significant indirect effect of change in temperature via voter turnout on voting results hints at the possibility that anger is also involved in voting behavior, though future studies are needed to fully take into account the role of anger and other emotions^3^. In this respect, it is noteworthy that smaller, non-system parties gain votes whereas the challenger, mainstream party does not (Van Zomeren et al., 2016). However, increased temperatures are unlikely to lead to system change during elections because the negative indirect effect of temperature for the incumbent party is fully compensated by its even stronger, positive direct effect. This result indicates that higher temperatures make the majority of voters increasingly lenient toward the party in power. Such findings are consistent with a ‘good weather effect’ (Cunningham, 1979; Guéguen and Lamy, 2013) and are reminiscent of the finding that good weather leads to a more favorable judgment of the government (Mutz and Kampfer, 2011).

The present findings also add to the literature of non-ideological and even non-rational factors that influence voting behavior. The theme of (ir)rationality has been linked to citizens’ decision to vote (Downs, 1957; Geys, 2006; Caplan, 2007), as well as to the basis of the particular preferences of voters. For instance, people base their vote on the candidates’ facial features (Todorov et al., 2005; Antonakis and Dalgas, 2009) and height (Simonton, 1994), rather than on the policy issues. Moreover, citizens often have limited knowledge and unstable attitudes about policy issues (Converse, 1964). The present demonstration of temperature effects adds to this literature, although future studies could expand our understanding by tapping into smaller levels of analyses (e.g., cities or counties).

One could rightfully argue that although the effect of temperature on voting is significant, its effect size is relatively small. However, two examples of American presidential elections illustrate that even small shifts in votes may be consequential in close races. In 1960, the 35th President of the United States, John F. Kennedy, earned 49.72% of the votes whereas the incumbent party’s candidate Richard M. Nixon earned 49.55% of the votes, a difference of only 0.17%. A closer look at the results of this election reveals that Kennedy had a slightly higher share of votes than Nixon in Hawaii (0.06%), Illinois (0.18%), and Missouri (0.52%). If Nixon had won the latter states, he would have become president. Another example concerns the 2000 presidential election. Based on our model, an increase of only 1°C (1.8°F) may have made Al Gore the 43rd United States President instead of George W. Bush, as Gore would have won in Florida. It is often mentioned that “the heat is on” during presidential campaigns, and our findings indeed clarify that temperature matters when it comes to actual voting.

## Author Contributions

JVA and AVH developed the study concept and design. Data collection was performed by JS. Data analysis and interpretations was conducted by JVA under the supervision of AVH. JVA and AVH drafted the manuscript, and BB, DDC, and AR provided critical revisions. All authors approved the final version of the manuscript for submission.

## Conflict of Interest Statement

The authors declare that the research was conducted in the absence of any commercial or financial relationships that could be construed as a potential conflict of interest.

## Footnotes

1 We are only aware of three studies which included absolute temperature (instead of change in temperature). Eisinga et al. (2012) reported that elections taking place in hotter entities are accompanied by greater turnout. Other studies (Gomez et al., 2007; Artés, 2014) reported non-significant effects.

2 The asterisk sign (‘^∗^’) indicates multiplication.

3 In the American National Election Studies (ANES) datasets, a single-item measure of angry feelings toward the incumbent president was administered from 1980 onward (but not always in each state). We aggregated the available individual-level data into a state-level measure of anger per election year (*N* = 248 data points, i.e., 33% of our original dataset), and then calculated the relative change. Increases in anger correlated with increases in voter turnout, increases in votes for the challenger party, and decreases in votes for the incumbent party. Moreover, the effects of voter turnout on voting behavior dropped considerably once anger was taken into account, signifying that at least part of its effects can be explained by anger. The full results of these analyses can be found in the Supplementary Materials.

## References

[B1] Anderson C. A. Bushman B. J. (2002). The general aggression model: an integrated social-cognitive model of human aggression. *Annu. Rev. Psychol.* 53 27–51. 10.1146/annurev.psych.53.100901.135231 11752478

[B2] Antonakis J. Dalgas O. (2009). Predicting elections: child’s play! *Science* 323 1183 10.1126/science.1167748 19251621

[B3] Artés J. (2014). The rain in Spain: turnout and partisan voting in Spanish elections. *Eur. J. Polit. Econ.* 34 126–141. 10.1016/j.ejpoleco.2014.01.005

[B4] Bushman B. J. Wang M. C. Anderson C. A. (2005). Is the curve relating temperature to aggression linear or curvilinear? Assaults and temperature in Minneapolis reexamined. *J. Pers. Soc. Psychol.* 89 62–66. 10.1037/0022-3514.89.1.62 16060743

[B5] Caplan B. (2007). *The Myth of the Rational Voter: Why Democracies Choose Bad Policies*. Princeton, NJ: Princeton University Press.

[B6] Carlsmith J. M. Anderson C. A. (1979). Ambient temperature and the occurrence of collective violence: a new analysis. *J. Pers. Soc. Psychol.* 37 337–344. 10.1037/0022-3514.37.3.337 458551

[B7] Converse P. E. (1964). “The nature of belief systems in mass publics,” in *Ideology and Discontent* ed. Apter D. E. (New York, NY: Free Press) 206–261.

[B8] Cunningham M. R. (1979). Weather, mood, and helping behavior: quasi experiments with the sunshine Samaritan. *J. Pers. Soc. Psychol.* 31 1947–1956. 10.1037/0022-3514.37.11.1947

[B9] Curriero F. C. Heiner K. S. Samet J. M. Zeger S. L. Strug L. Patz J. A. (2002). Temperature and mortality in 11 cities of the eastern United States. *Am. J. Epidemiol.* 155 80–87. 10.1093/aje/155.1.80 11772788

[B10] Downs A. (1957). *An Economic Theory of Democracy.* New York, NY: Harper and Row.

[B11] Eisinga R. Te Grotenhuis M. Pelzer B. (2012). Weather conditions and voter turnout in Dutch national parliament elections, 1971-2010. *Int. J. Biometeorol.* 56 783–786. 10.1007/s00484-011-0477-7 21792567 PMC3382632

[B12] Foster C. A. Witcher B. S. Campbell W. K. Green J. D. (1998). Arousal and attraction: evidence for automatic and controlled components. *J. Pers. Soc. Psychol.* 74 86–101. 10.1037/0022-3514.74.1.86

[B13] Geys B. (2006). ‘Rational’ theories of voter turnout: a review. *Polit. Stud. Rev.* 4 16–35. 10.1111/j.1478-9299.2006.00034.x

[B14] Gomez B. T. Hansford T. G. Krause G. A. (2007). The Republicans should pray for rain: weather, turnout, and voting in U.S. Presidential Elections. *J. Polit.* 69 649–663. 10.1111/j.1468-2508.2007.00565.x

[B15] Guéguen N. Lamy L. (2013). Weather and helping: additional evidence on the effect of the sunshine Samaritan. *J. Soc. Psychol.* 153 123–126. 10.1080/00224545.2012.720618 23484341

[B16] Kusuda T. Achenbach P. R. (1965). *Earth Temperature and Thermal Diffusivity at Selected Stations in the United States (No. NBS-8972).* Gaithersburg, MD: National Bureau of Standards.

[B17] Lombroso C. (1911). *Crime: Its Causes and Remedies.* Boston, MA: Little, Brown.

[B18] Mutz M. Kampfer S. (2011). And now the weather: does weather influence the assessment of political and economical issues? *Z. Soziol.* 40 208–226.

[B19] Oishi S. (2014). Socioecological psychology. *Annu. Rev. Psychol.* 65 581–609. 10.1146/annurev-psych-030413-152156 23987114

[B20] Raudenbush S. W. Bryk A. S. (2002). *Hierarchical Linear Models: Applications and Data Analysis Methods* 2nd Edn. Thousand Oaks, CA: Sage.

[B21] Schwartz D. C. (1968). On the ecology of political violence: “The long hot summer” hypothesis. *Am. Behav. Sci.* 11 24–28. 10.1177/000276426801100607

[B22] Simonton D. K. (1994). *Greatness: Who Makes History and Why.* New York, NY: Guilford Press.

[B23] Todorov A. Mandisodza A. N. Goren A. Hall C. C. (2005). Inferences of competence from faces predict election outcomes. *Science* 308 1623–1626. 10.1126/science.1110589 15947187

[B24] Valentino N. A. Brader T. Groenendyk E. W. Gregorowicz K. Hutchings V. L. (2011). Election night’s alright for fighting: the role of emotions in political participation. *J. Polit.* 73 156–170. 10.1017/S0022381610000939

[B25] Van Zomeren M. (2016). Building a tower of Babel? Integrating core motivations and features of social structure into the political psychology of action. *Polit. Psychol.* 37 87–114. 10.1111/pops.12322

[B26] Van Zomeren M. Susilani N. Berend S. (2016). Explaining a rare null relationship between group identification and social protest through a relational form of coping. *J. Soc. Polit. Psychol.* 4 381–402. 10.5964/jspp.v4i1.419

[B27] Zillmann D. (2003). “Theory of affective dynamics: emotions and moods,” in *Communication and Emotion: Essays in Honor of Dolf Zillmann* eds Bryant J. Roskos-Ewoldsen D. Cantor J. (Mahwah, NJ: Lawrence Erlbaum Associates) 533–567.

