# Supplementary material for: When the Heat Is On: The Effect of Temperature on Voter Behavior in Presidential Elections
Source: Front Psychol. 2017 Jun 8;8:929. doi: 10.3389/fpsyg.2017.00929 (PMC5463178; doi:10.3389/fpsyg.2017.00929)
Supplement: Supplementary file 1 [file Table_1.DOCX]

When the Heat is On: The Effect of Temperature on Voter Behavior in Presidential Elections

**Supplementary Materials**

We collected data from U.S. presidential elections from 1960 to 2016 in each State (and in Washington D.C.). Specifically, these elections were organized on the following dates (MM.DD/YYYY), with the winner of these elections in parentheses:

08.11.1960 (J. F. Kennedy), 03.11.1964 (L. B. Johnson), 05.11.1968 (R. M. Nixon), 07.11.1972 (R. M. Nixon), 02.11.1976 (J. E. Carter), 04.11.1980 (R. W. Reagan), 06.11.1984 (R. W. Reagan), 08.11.1988 (G. H. W. Bush), 03.11.1992 (B. W. J. Clinton), 05.11.1996 (B. W. J. Clinton), 07.11.2000 (G. W. Bush), 02.11.2004 (G. W. Bush), 04.11.2008 (B. H. Obama), 06.11.2012 (B. H. Obama), and 08.11.2016 (D. J. Trump).

All elections were held in each State (15 * 50 elections = 750 data points) and all elections, except the 1960 election, were held in Washington D. C. (14 elections = 14 data points; D.C. was only enfranchised for presidential election through the 23^rd^ Amendment in 1961).

The temperature data were retrieved from the web application of the National Centers for Environmental Information (<http://www.ncdc.noaa.gov/cdo-web/search?datasetid=GHCND>). We selected a weather station close to center of population for each state (e.g., for Colorado, see <https://en.wikipedia.org/wiki/Colorado#Demographics> and <https://en.wikipedia.org/wiki/Colorado#/media/File:Colorado_population_map.png>). In case of missing data, we sought the nearest station, and in all cases there was available a neighboring station within a close distance. Not only maximum temperature on Election Day was retrieved, but also the maximum temperature for the seven days preceding the elections were collected.

Data of the following weather stations were analyzed:

Table 1. Weather stations per state.

| Alabama | Selma |
| --- | --- |
| Alaska | Anchorage, Matanuska Agricultural Experiment Station |
| Arizona | Casa Grande National Monument |
| Arkansas | Pine Bluff |
| California | Riverside Fire Station 3; Clark California |
| Colorado | Fort Collins |
| Connecticut | Hartford Brainard Field |
| D. C. | National Arboretum, Dalecarlia Reservoir |
| Delaware | Dover, Wilmington Nev. |
| Florida | Bartow |
| Georgia | Hawkinsville |
| Hawaii | Honolulu International Airport |
| Idaho | Oakley |
| Illinois | Aurora |
| Indiana | Whitestown |
| Iowa | Perry |
| Kansas | Herington |
| Kentucky | Bluegrass Airport |
| Louisiana | Alexandria |
| Maine | Gardiner |
| Maryland | Laurel 3 W, Baltimore International Airport |
| Massachusetts | East Milton Blue Hill Observatory |
| Michigan | Owosso WWTP |
| Minnesota | Zumbrota, Faribault |
| Mississippi | Kosciusko |
| Missouri | Fulton |
| Montana | Bozeman Montana SU |
| Nebraska | Columbus 3 |
| Nevada | Fallon Experiment Station |
| New Hampshire | Durham |
| New Jersey | Hightstown 2 W |
| New Mexico | Albuquerque International Airport |
| New York | NY Central Park Obs. Belvedere Tower |
| North Carolina | Albemarle |
| North Dakota | MC Clusky, Turtle Lake |
| Ohio | Circleville |
| Oklahoma | Shawnee, Chandler OK, Guthrie Municip. |
| Oregon | Salem McNary Field |
| Pennsylvania | York 3 SSW Pump Station |
| Rhode Island | Kingston |
| South Carolina | Winnsboro |
| South Dakota | Miller SD |
| Tennessee | Murfreesboro |
| Texas | Hillsboro |
| Utah | Logan UT State University |
| Vermont | Rutland, Cornwall |
| Virginia | Hopewell |
| Washington | Sedro Woolley |
| West Virginia | Spencer |
| Wisconsin | Portage |
| Wyoming | Cheyenne Municipal Airport |

Election results were retrieved from <http://uselectionatlas.org/RESULTS/> and <https://en.wikipedia.org/wiki/United_States_presidential_election>. Like most election studies with American samples, we used Voting Age (VAP) Population as the denominator (see, B. Geys, *Elect Stud*, **25,** 637-663. (2006). Voter turnout was calculated by the following formula: 100% - (VAP – number of votes)/VAP]. VAP per State was retrieved from: <http://uselectionatlas.org/RESULTS/>. We wanted to avoid detecting spurious correlations by including as many relevant control variables as possible. In detail, we included regional coordinates to control for regional cultural differences (e.g., culture of honor, see Rentfrow, 2010; see also Curriero et al., 2002), which might potentially drive voting behavior. We included absolute temperature to control for regional temperature differences. Logically, voter turnout and voting behaviors can also depend on whether the president is re-electable, whether the president can be impeached, whether the president’s party has a majority in the congress, and the approval rating of the incumbent president (see also Abramowitz & Stone, 2006; Blais, 2008). Presidential approval ratings were retrieved from: <http://www.gallup.com/poll/116677/Presidential-Approval-Ratings-Gallup-Historical-Statistics-Trends.aspx>. Finally, we included state-level changes in GDP as economic conditions might also influence voters (e.g., Fiorina, 1978), and there is robust evidence for the claim that “good times keep parties in office, bad times cast them out” (Lewis-Beck & Stegmeier, 2000).

Table 2 reports the changes in model fit for the multilevel models. The step-by-step addition of all blocks of predictors improved the goodness-of-fit statistics of each multilevel model significantly, except for the model predicting change in votes for the challenger party.

Table 2. Changes in Model Fit (i.e., Δ Deviance provided by χ²(df = 1) change in -2 * log-likelihood) in Multilevel Hierarchical Regression Steps.

|  | Δ Turnout | Δ Votes for non-system parties | Δ Votes for Challenger party | Δ Votes for Incumbent party |
| --- | --- | --- | --- | --- |
| Step 0 – 1 | 713.82^***^ | 430.71^***^ | 881.94^***^ | 922.58^***^ |
| Step 1 – 2 | 5.64^*^ | 25.13^***^ | 0.00 | 21.79^***^ |
| Step 2 – 3 |  | 52.27^***^ | 0.29 | 45.00^***^ |

Note: ^*^: *p* < .05; ^**^: *p* < .01; ^***^: *p* < . 001
For all outcomes, in Step 1, state coordinates and absolute temperature indicators, presidential and incumbent party indicators, and change in state Gross Domestic Product (GDP) are regressed upon the outcome. Step 2 explores the additional role of relative temperature compared to the previous election. For Δ Votes outcomes, an additional Step 3 explores the additional role of change in turnout compared to the previous election.

Table 3, 4, and 5 report analyses using data from the American National Election Studies (ANES) datasets. In these datasets, a single-item measure of angry feelings towards the incumbent president was administered from 1980 onwards (but not always in each state). We aggregated the available individual-level data (*N* = 35,797 respondents) into a state-level measure of anger per election year (*N* = 248 data points, i.e., only 33% of our original dataset), and then calculated the relative change with regards to the previous election. Table 3 shows the unstandardized estimates of the multilevel regression analyses on the respective outcomes when no control variables were included. Tables 4 and 5 report the analyses on voter turnout and voting behavior, respectively, when control variables were included.

Table 3. Unstandardized Estimates (Standard Errors in parentheses) of Multilevel Hierarchical Regression Analyses on Change in Voter Turnout and Voting Result.

|  | Voter turnout | Non-system parties | Challenger party | Incumbent party |
| --- | --- | --- | --- | --- |
| Predictor | *b*  (*SE*) | *b*  (*SE*) | *b*  (*SE*) | *b*  (*SE*) |
| Δ State anger towards president | 4.58^*^  (1.94) | -1.02  (1.96) | 4.19^***^  (1.06) | -3.15^a^  (1.71) |

Note: ^a^: *p* < .05, one tailed; *: *p* < .05; ***: *p* < .001

Table 4. Unstandardized Estimates (Standard Errors in parentheses) of Multilevel Hierarchical Regression Analyses on Change in Voter Turnout.

| Predictor | *b*  (*SE*) |  |
| --- | --- | --- |
| Latitude | 0.29^a^  (0.17) |  |
| Longitude | -0.05  (0.03) |  |
| Temperature Election Day | 0.00  (0.15) |  |
| Temperature week before Election Day | 0.39^*^  (0.17) |  |
| President eligible for reappointment (1=yes) | -1.11^*^  (0.51) |  |
| Approval rating incumbent president | 0.25^***^  (0.07) |  |
| Majority in Congress in last 2 years (1=yes) | 2.82^***^  (0.84) |  |
| Δ State GDP per capita | -28.19^***^  (6.57) |  |
| Δ Temperature on Election Day | 0.22^*^  (0.09) |  |
| Δ State anger towards incumbent president | 4.45^**^  (1.71) |  |

Note: GDP = gross domestic product; ^a^: *p* < .05, one tailed *: *p* < .05; **: *p* < .01; ***: *p* < .001. The parameter “the incumbent president being elected or not” is set to zero because it is redundant in these analyses (i.e., its value is always 0 due to the few number of observations).

Table 5. Unstandardized Estimates (Standard Errors in parentheses) of Multilevel Hierarchical Regression Analyses on change in votes for Alternative Parties (Independent, Libertarians, Greens), the Challenger Mainstream Party and the Incumbent Party.

|  | Non-system parties | | | Challenger party | | | Incumbent party | | |
| --- | --- | --- | --- | --- | --- | --- | --- | --- | --- |
| Predictor | *b*  (*SE*) |  |  | *b*  (*SE*) |  |  | *b*  (*SE*) |  |  |
| Latitude | 0.11  (0.19) |  |  | -0.06  (0.09) |  |  | -0.05  (0.16) |  |  |
| Longitude | -0.01  (0.03) |  |  | 0.04^*^  (0.02) |  |  | -0.03  (0.03) |  |  |
| Temperature Election Day | 0.49^**^  (0.16) |  |  | -0.09  (0.08) |  |  | -0.40^**^  (0.14) |  |  |
| Temperature week before | -0.45^*^  (0.19) |  |  | -0.02  (0.09) |  |  | 0.47^**^  (0.16) |  |  |
| President eligible for reappointment | -1.81^***^  (0.56) |  |  | -2.99^***^  (0.28) |  |  | 1.17^*^  (0.48) |  |  |
| Approval rating | 0.34^***^  (0.08) |  |  | 0.01  (0.04) |  |  | -0.35^***^  (0.07) |  |  |
| Majority in Congress | -4.80^***^  (0.94) |  |  | 0.63  (0.46) |  |  | -4.18^***^  (0.81) |  |  |
| Δ State GDP per capita | -15.99^*^  (7.49) |  |  | -5.91  (3.64) |  |  | 21.72^***^  (6.43) |  |  |
| Δ Temperature on Election Day | -0.33^**^  (0.11) |  |  | 0.10^a^  (0.07) |  |  | 0.23^*^  (0.09) |  |  |
| Δ Voter Turnout | 0.31^***^  (0.07) |  |  | -0.16^***^  (0.03) |  |  | -0.15^*^  (0.06) |  |  |
| Δ State anger towards president | -3.17^a^  (1.91) |  |  | 1.47  (0.93) |  |  | 1.70  (1.64) |  |  |

Note: GDP = gross domestic product; ^a^: *p* < .05, one tailed; *: *p* < .05; **: *p* < .01; ***: *p* < .001. The parameter “the incumbent president being elected or not” is set to zero because it is redundant in these analyses (i.e., its value is always 0 due to the few number of observations).
